# Bortezomib inhibits growth and sensitizes glioma to temozolomide (TMZ) via down-regulating the FOXM1–Survivin axis

**DOI:** 10.1186/s40880-019-0424-2

**Published:** 2019-12-03

**Authors:** Jun-Hai Tang, Lin Yang, Ju-Xiang Chen, Qing-Rui Li, Li-Rong Zhu, Qing-Fu Xu, Guo-Hao Huang, Zuo-Xin Zhang, Yan Xiang, Lei Du, Zheng Zhou, Sheng-Qing Lv

**Affiliations:** 1 Department of Neurosurgery, Xinqiao Hospital, Third Military Medical University, Chongqing, 400037 P. R. China; 2 Department of Neurosurgery, Changzheng Hospital and Shanghai Institute of Neurosurgery, Second Military Medical University, Shanghai, 200003 P. R. China; 3 Institute of Pathology and Southwest Cancer Center, Southwest Hospital, Third Military Medical University, Chongqing, 400038 P. R. China; 4 Department of Ultrasound, Children Hospital, Chongqing Medical University, Chongqing, 400010 P. R. China; 5 Department of Neurosurgery, The Second Xiangya Hospital, Central South University, Changsha, 410008 Hunan P. R. China

**Keywords:** Glioma, Bortezomib, *FOXM1*, *Survivin*, Temozolomide (TMZ), Chemotherapy

## Abstract

**Background:**

High-grade glioma (HGG) is a fatal human cancer. Bortezomib, a proteasome inhibitor, has been approved for the treatment of multiple myeloma but its use in glioma awaits further investigation. This study aimed to explore the chemotherapeutic effect and the underlying mechanism of bortezomib on gliomas.

**Methods:**

U251 and U87 cell viability and proliferation were detected by 3-(4,5-dimethyl-2-thiazolyl)-2,5-diphenyl-2-*H*-tetrazolium bromide (MTT) assay, tumor cell spheroid growth, and colony formation assay. Cell apoptosis and cell cycle were detected by flow cytometry. Temozolomide (TMZ)-insensitive cell lines were induced by long-term TMZ treatment, and cells with stem cell characteristics were enriched with stem cell culture medium. The mRNA levels of interested genes were measured via reverse transcription-quantitative polymerase chain reaction, and protein levels were determined via Western blotting/immunofluorescent staining in cell lines and immunohistochemical staining in paraffin-embedded sections. Via inoculating U87 cells subcutaneously, glioma xenograft models in nude mice were established for drug experiments. Patient survival data were analyzed using the Kaplan–Meier method.

**Results:**

Bortezomib inhibited the viability and proliferation of U251 and U87 cells in a dose- and time-dependent manner by inducing apoptosis and cell cycle arrest. Bortezomib also significantly inhibited the spheroid growth, colony formation, and stem-like cell proliferation of U251 and U87 cells. When administrated in combination, bortezomib showed synergistic effect with TMZ in vitro and sensitized glioma to TMZ treatment both in vitro and in vivo. Bortezomib reduced both the mRNA and protein levels of Forkhead Box M1 (*FOXM1*) and its target gene *Survivin*. The FOXM1–Survivin axis was markedly up-regulated in established TMZ-insensitive glioma cell lines and HGG patients. Expression levels of FOXM1 and Survivin were positively correlated with each other and both related to poor prognosis in glioma patients.

**Conclusions:**

Bortezomib was found to inhibit glioma growth and improved TMZ chemotherapy efficacy, probably via down-regulating the FOXM1–Survivin axis. Bortezomib might be a promising agent for treating malignant glioma, alone or in combination with TMZ.

## Background

High-grade glioma (HGG) is one of the leading causes of cancer mortality in adults and impose a great challenge on its treatment [1, 2]. Owing to the introduction of the alkylating agent temozolomide (TMZ) and the adoption of radiotherapy with concomitant and adjuvant TMZ treatment, the median survival of patients with glioblastoma multiforme (GBM) has been prolonged from 12.1 to 14.6 months [3]. However, the overall clinical effect of this regimen is still disappointing, mostly due to the inherent or induced resistance to TMZ therapy [4]. Thus, more comprehensive understanding of the progression and resistance mechanisms and novel therapeutic targets are urgently needed for the clinical management of this fatal tumor [5].

The ubiquitin–proteasome system plays an important role in the regulation of cell growth and survival, and the 26S proteasome is an essential component for degrading 80–90% of dysfunctional proteins and preventing their intracellular accumulation [6, 7]. Tumor cells are characterized by uncontrolled proliferation and rapidly accumulation of abnormal proteins, and timely degradation of these substrates is essential for cancer cell growth and survival. In accordance, aberrant activation of the proteasome has been widely observed in various types of cancers and implicated in the development and progression of carcinogenesis [8]. However, this highly dependence upon proteasome activity makes tumorigenic cells more sensitive to proteasomal inhibition than normal cells, contributing to specific targeting of tumor cells by proteasome inhibitors (PIs) [9, 10]. Bortezomib (PS-341/Velcade) was the first US Food and Drug Administration (FDA) approved PI used in the treatment of newly diagnosed multiple myeloma, relapsed/refractory multiple myeloma, and mantle cell lymphoma [11]. The FDA approval of bortezomib for the treatment of multiple myeloma provided a “proof of concept” that targeting the ubiquitin–proteasome pathway was a viable route for cancer treatment. Accumulating studies have shown that bortezomib was an active antitumor agent in a variety of solid malignancy models, both in vitro and in vivo [12, 13].

Aberrant high proteasomal activity was also found in glioma cells, especially in the group resembling glioma stem cells (GSCs), suggesting that bortezomib could be a potential chemotherapeutic agent for malignant gliomas [14]. Although few previous studies have indicated the killing effect of bortezomib on glioma cells [14, 15], more comprehensive investigations on the chemotherapeutic role of bortezomib in glioma treatment as well as the related molecular mechanism are still in urgent need. As such, in this study, we initially treated U251 and U87 cell lines with different concentrations of bortezomib and determined the consequent alterations in cell viability, proliferation, apoptosis, cell cycle distribution, colony formation, and stem cell characteristics. We then explored the effects of bortezomib on the efficacy of TMZ chemotherapy by testing bortezomib and TMZ combined treatment in both cell lines and glioma xenograft models. In our study, the “FOXM1–Survivin” axis was supposed to be an important target of bortezomib. So, we investigated the regulatory role of bortezomib on the FOXM1–Survivin axis with in vitro cell lines and in vivo xenograft models. In addition, we also measured the expression level of the FOXM1–Survivin axis in clinical glioma samples and analyzed its relation with patient prognosis. We expect that our findings would help, to a certain extent, to clarify the chemotherapeutic effect and underlying mechanism of bortezomib treatment on gliomas.

## Materials and methods

### Cell lines and cell culture

Human glioblastoma-derived U87, U251, LN229, A172, SF295, and astrocytoma-derived SF268 cell lines were obtained from the American Type Culture Collection (ATCC, Manassas, VA, USA, passages 5-15), verified for purity using the ATCC cell line authentication service, and routinely tested for mycoplasma. All cell lines were cultured in Dulbecco’s modified Eagle’s medium (DMEM, Gibco, Grand Island, NY, USA) supplemented with 10% fetal bovine serum (Gibco) and 1% penicillin/streptomycin (Invitrogen, Carlsbad, CA, USA). All cells were cultured under 37 °C in a humidified atmosphere with 5% CO_2_.

### Clinical glioma samples

This study was reviewed and approved by the Ethics Committee of the Third Military Medical University, Chongqing, China. Informed consents were obtained from all patients or his/her guardians. Clinical specimens [10 para-tumor brain tissues, 10 World Health Organization (WHO) grade I–II gliomas, 10 WHO grade III gliomas and 10 WHO grade IV GBM] were obtained from glioma patients who underwent surgery at the Department of Neurosurgery, Xinqiao Hospital between February 2014, and August 2019. All tumor samples were histologically confirmed as brain glioma by at least two experienced pathologists. All involved patients were 18 to 75 years old, had detailed clinical history and follow-up information, and had no prior radiotherapy to the brain and no intracranial abscess within 6 months before surgery. The baseline clinical information of glioma patients are summarized in Table 1. Online data of glioma patients were downloaded from The Cancer Genome Atlas (TCGA) Research Network (https://www.cancer.gov/tcga) [16].

**Table 1:** Baseline clinical information of the investigated glioma patients

Variable	Number of cases
Gender	
Male	14
Female	16
WHO grade	
I–II	10
III	10
IV	10
Age (years)	
< 40	7
40–49	10
50–59	9
60–69	2
≥ 70	2
Resection type	
Total	24
Partial	6
Survival status	
Alive	17
Dead	13
Pathologic diagnosis	
Astrocytoma	7
Oligodendroglioma/oligoastrocytoma	3
Anaplastic astrocytoma	5
Anaplastic oligodendroglioma/mixed	5
Glioblastoma	10

*WHO* World Health Organization

### MTT assay

Exponentially growing cell lines were digested and seeded into 96-well plates with 4 × 10^3^ cells/well. After treatment with different concentrations of bortezomib (Selleck Chemicals, Houston, TX, USA), TMZ (Selleck Chemicals), or their combination, cell viability was detected. After adding 20 µL MTT reagent (5 mg/mL) in each well and another 4 h normal culture, the medium was carefully removed, and 100 µL formazan solution was added in each well. The optical density (OD) was measured at 570 nm using an Ultra Multi-functional Microplate Reader (Tecan, Durham, NC, USA). Cell proliferation inhibition rates and survival rates were used to represent the inhibiting effect of different treatments on cell viability, and they were calculated using the following formulae: cell proliferation inhibition rate = 100% × [mean OD value of control group − mean OD value of treatment group]/mean OD value of control group; cell survival rate = 100% × [mean OD value of treatment group/mean OD value of control group]. The 50% inhibitory concentration (IC_50_) of drug used was calculated with the method of “log(inhibitor) vs. normalized response-Variable slope” using GraphPad Prism 7.0 (GraphPad Software, San Diego, CA, USA). Quantitative analysis of dose–effect relationships and calculation of combination index were performed by CompuSyn (ComboSyn, Inc., Paramus, NJ, USA).

### Colony formation assay

Glioma cells were seeded into 6-well culture plate with 200 cells/well and cultured for 10 days. Colonies were washed with cold phosphate buffer saline (PBS) and fixed with 4% paraformaldehyde. Images were taken on a digital microscope (OLYMPUS, Ishikawa, Japan). Those colonies composed of more than 15 cells were counted manually. The number of colonies was represented by the average number from five random fields.

### Tumor cell spheroid assay, enrichment of cells with GSC characteristics, and induction of TMZ-insensitive cell lines

Exponentially growing cells were digested and added into a U-bottom 96-well plate at a concentration of 1 × 10^3^ cells/well in 100 μL medium. After centrifuging at 1000×*g* for 5–10 min, the cells were cultured for another 24 h. The top half medium was carefully replaced with fresh medium containing drug at day 1, and with normal medium at days 4 and 8. Images of spheroids were taken every 2 days. The surface (superficial) area of spheroids on planar images was used to represent the size of real spheroids and was measured using the Image-pro Plus 6.0 (Media Cybernetics, Rockville, MD, USA). The medium for stem cell culture was composed of 20 ng/mL epidermal growth factor, 20 ng/mL basic fibroblast growth factor, 1% N-2 supplement (500×), 1% Glutamax, 0.2% heparin, and 1% penicillin/streptomycin in DMEM/F12ham. After culturing for 24 h with normal medium with or without bortezomib, the cells were digested and seeded into 6-well plates with 2 × 10^3^ cells/well in 1 mL stem cell culture medium. 500 μL fresh stem cell culture medium was added every 3 days. Images were taken every 2 days. To induce TMZ-insensitive U251 and U87 cell lines, U251 and U87 cells were cultured in 10-cm dishes under a 10-day insensitivity-inducing process with normal medium at days 1, 2, 6, and 7, and with medium containing 200 or 500 μmol/L TMZ at days 3, 4, 5, 8, 9, and 10. The process was conducted for at least 3 cycles. Digestion and splitting were conducted when tumors cells reached 100% confluence in one dish.

### Flow cytometry detecting cell apoptosis and cell cycle

Cell apoptosis and cell cycle were detected with the Annexin V-FITC Apoptosis Detection Kit (C1062S, Beyotime Biotechnology, Shanghai, China) and the Cell Cycle and Apoptosis Analysis Kit (C1052, Beyotime Biotechnology), performed according to the manufacturer’s instructions [17]. Cell apoptosis and cell cycle were measured and analyzed by a flow cytometry machine (FACS Calibur™, BD Biosciences, San Jose, CA, USA).

### Lentivirus packaging

The culture medium of 85% confluent 293T cells was replaced with Opti-MEM 2 h before plasmid transfection. Using Lipofectamine 2000, we initially transfected 293T cells with a Lenti-easy packaging mix and *FOXM1* overexpression (OE) plasmid (GV270-*FOXM1*) or control empty vector (EV) (GeneChem, Shanghai, China). After transfection for 8 h, the cell medium was replaced with normal medium and cultured for another 48 h. Lentivirus particles were collected from cell medium by centrifuging (1500 rpm for 5 min) and filtering (0.22 μm) processes.

### Stable overexpression and transient knockdown of *FOXM1*

For overexpressing *FOXM1*, 50% confluent U251 and U87 cells in 6-well plates were cultured in 1 mL medium with lentivirus particles and 5 μg/mL polybrene (GeneChem). 12 h later, the cell medium was replaced with 2 mL fresh normal medium and cultured for another 48 h. Medium with 2 μg/mL puromycin was used for selecting stably transfected cells, and this puromycin-containing medium was refreshed every 3 days for at least a 9-day selection process. For *FOXM1* transient knockdown, 50% confluent U251, U87, and LN229 cells were transfected with *FOXM1*-short interfering RNA (siRNA) oligonucleotide (CUCUUCUCCCUCAGAU AUAdTdT) or control siRNA (RiboBio, Guangzhou, Guangdong, China). After transfection for 12 h, fresh normal medium was added, and cells were cultured for another 48 h before performing the following experiments.

### Reverse transcription-quantitative polymerase chain reaction (RT-qPCR)

Total RNA from cultured cells or frozen glioma tissues was extracted using the Trizol reagent (Invitrogen). First-strand complementary DNA (cDNA) was reversely transcribed from 1 μg total RNA using the Prime Script™ RT Master Mix (Code No. RR047A, Takara Bio, Shiga, Japan). Target gene mRNA was amplified with SYBR^®^ Premix Ex Taq™ II kit (Code No. RR820A, Takara Bio) and measured by CFX96™ Real-time System (Bio-Rad, Irvine, CA, USA). Each single reaction system (15 µL) consisted of 7.50 µL 2× KAPA SYBR Fast qPCR Master Mix Universal, 0.15 µL forward primer (10 µmol/L), 0.15 µL reverse primer (10 µmol/L), 1.00 µL (50.00 ng) cDNA template and 6.20 µL PCR grade water. Glyceraldehyde-3-phosphate dehydrogenase (GAPDH) was used as an internal control and genes’ specific primers are presented in Table 2.

**Table 2:** Genes’ specific primers used for RT-qPCR

Gene	Primer sequence
*GADPH*	F 5′- GACCCCTTCATTGACCTCAAC-3′
	R 5′-TGGACTGTGGTCATGAGTCC-3′
*FOXM1*	F 5′-CGTCGGCCACTGATTCTCAAA-3′
	R 5′-GGCAGGGGATCTCTTAGGTTC-3′
*Survivin*	F 5′-AGGACCACCGCATCTCTACAT-3′
	R 5′-AAGTCTGGCTCGTTCTCAGTG-3′
*Nestin*	F 5′-CACCTGTGCCAGCCTTTCTTA-3′
	R 5′-TTTCCTCCCACCCTGTGTCT-3′
*SOX2*	F 5′-CAAGATGCACAACTCGGAGA-3′
	R 5′-GCTTAGCCTCGTCGATGAAC-3′
*Oct4*	F 5′-CTGGAGAAGGAGAAGCTGGA-3′
	R 5′-CAAATTGCTCGAGTTCTTTCTG-3′

RT-qPCR, reverse transcription-quantitative polymerase chain; *GADPH*, glyceraldehyde-3-phosphate dehydrogenase; *FOXM1*, Forkhead Box M1; *Survivin*, *BIRC5*, baculoviral IAP repeat containing 5; *SOX2*, SRY-box transcription factor 2; *Oct4*, *POU5F1*, POU class 5 homeobox 1

### Western blotting

Total protein was extracted with RIPA buffer, and 35–50 μg samples were loaded after measuring their concentration using a Pierce BCA kit (Thermo Fisher Scientific, Waltham, MA, USA) and separated by 7.5% or 10% sodium dodecyl sulfate–polyacrylamide gel electrophoresis. Polyvinylidene difluoride membranes were blocked with 5% fat-free milk for 1 h at room temperature and incubated in primary antibodies (Additional file 1: Table S1) at 4 °C overnight. After washing with tris buffered saline with Tween 200, the membranes were incubated in horseradish peroxidase (HRP)-conjugated anti-mouse or anti-rabbit secondary antibodies (Additional file 1: Table S1) for 1 h at room temperature. Protein bands were detected using the enhanced chemiluminescence system (Thermo Fisher Scientific).

### Cellular immunofluorescent staining

Cells cultured in 24-well dishes were washed with cold PBS, fixed with 4% paraformaldehyde and permeabilized with 0.2% Triton X-100 (Sigma-Aldrich, St. Louis., MO, USA) for 10 min at room temperature. After blocking with 10% goat or donkey serum and washing, the cells were incubated with primary rabbit monoclonal antibodies to human FOXM1 and Survivin (Additional file 1: Table S1) at 4 °C overnight. The cells were incubated with Alexa Fluor488-conjugated secondary antibody (Additional file 1: Table S1) for 1 h at room temperature in the dark. Nuclei were counterstained with 4′,6-diamidino-2-phenylindole (DAPI) for 1 min. Fluorescence was visualized on a fluorescence microscope (FV-1000, OLYMPUS).

### Immunohistochemistry (IHC) analysis and scoring

Tissue samples were formalin-fixed and paraffin-embedded. Slides were deparaffinized and rehydrated via successive immersion in the following solutions: 100% xylene I (10 min), 100% xylene II (10 min), 100% ethanol I (5 min), 100% ethanol II (5 min), 95% ethanol (5 min), 85% ethanol (5 min), 80% ethanol (5 min), 75% ethanol (5 min), 80% ethanol (5 min), double distilled water I (10 min), and double-distilled water II (10 min). The slides were then boiled in 0.01 mol/L citrate buffer at 99 °C for 20 min, and endogenous peroxidase activity was blocked with 0.3% H_2_O_2_ in methanol for 30 min. Goat serum (Beyotime Biotechnology) was used to block the antibody at room temperature for 10 min. After overnight incubation at 4 °C in primary antibodies (Additional file 1: Table S1), the slides were exposed to HRP-labeled secondary antibodies (Additional file 1: Table S1) for 1 h at room temperature and developed with 3,3′-diaminobenzidine system. Staining intensity was accessed by a designated member of our group using the Image-pro Plus 6.0 (Media Cybernetics) and was represented by the mean density, using the formula mean density = integrated optical density/area of interest. Here, the IHC intensity of each slide was determined by the average “mean density” of at least 3 images from it, and the protein expression was assessed as “weak” (IHC intensity was 0 to 0.15), “moderate” (IHC intensity was 0.16 to 0.25), “strong” (IHC intensity was 0.26 to 0.35), and “very strong” (IHC intensity was above 0.35).

### Subcutaneous glioma xenograft model

All experiments involving mice were performed under the ethical criteria of the Third Military Medical University Animal Care and Use Committee, and guidelines for the Care and Use of Laboratory Animals (NIH publications Nos. 80-23, revised 1996) were seriously conducted during the whole process. To establish xenograft model of glioma in mice, 5 × 10^6^ human U87 cells suspended in 80 μL PBS were inoculated subcutaneously into the right hindlimb interior root of BALB/c nude mice (4-week old, female, purchased from Beijing Vital River Laboratory Animal Technology Co., Ltd., Beijing, China). About 5–8 days later, the mice bearing tumor around 50 mm^3^ were selected and randomized into a control group, bortezomib group, TMZ group or bortezomib + TMZ group. Mice in the control group received equivalent drug vehicle (PBS and dimethyl sulfoxide [DMSO]), mice in bortezomib group received 0.25 mg/kg bortezomib every 3 days (intraperitoneal injection [i.p.]), mice in TMZ group received 5 mg/kg TMZ on a 5 days on/2 days off regimen (4 cycles in total, i.p.), and mice in bortezomib + TMZ group received 0.25 mg/kg bortezomib every 3 days and also 5 mg/kg TMZ on a 5 days on/2 days off regimen. Tumor volume was measured every 3 days with a caliper and calculated using the formula tumor volume (mm^3^) = (length × width^2^)/2. About 28 days after the first treatment, all mice were euthanized, and the tumor bumps were carefully removed, weighed, and processed for IHC staining.

### Statistical analysis

The SPSS software, version 13.0 (SPSS, Inc., Chicago, IL, USA) and GraphPad Prism 7.0 (GraphPad Software) were used for statistical analysis. Data are presented as the mean ± standard deviation. The nonparametric unpaired *t* test was utilized to calculate the *P* value of the difference between 2 independent datasets. One-way analysis of variance (ANOVA) was used to analyze the significance among three or more independent datasets, and the Fisher’s Least Significant Difference method was used for multiple comparisons when the probability for ANOVA was statistically significant. Methods of nonparametric statistics such as the Mann–Whitney and Kruskal–Wallis tests were used when variances did not pass the Levene test for normality or homogeneity. For experiments with over 2 groups and repeated measurements at different time points, their data were processed by the repeated measure two-way ANOVA with Bonferroni post-test. Correlations of protein or mRNA expression between *FOXM1* and *Survivin* were performed using the Pearson *R* test. The Kaplan–Meier method was used to estimate survival rates. *P* < 0.05 was considered statistically significant.

## Results

### Bortezomib inhibited cell viability and induced apoptosis and cell cycle arrest in glioma cells

**Fig. 1:**
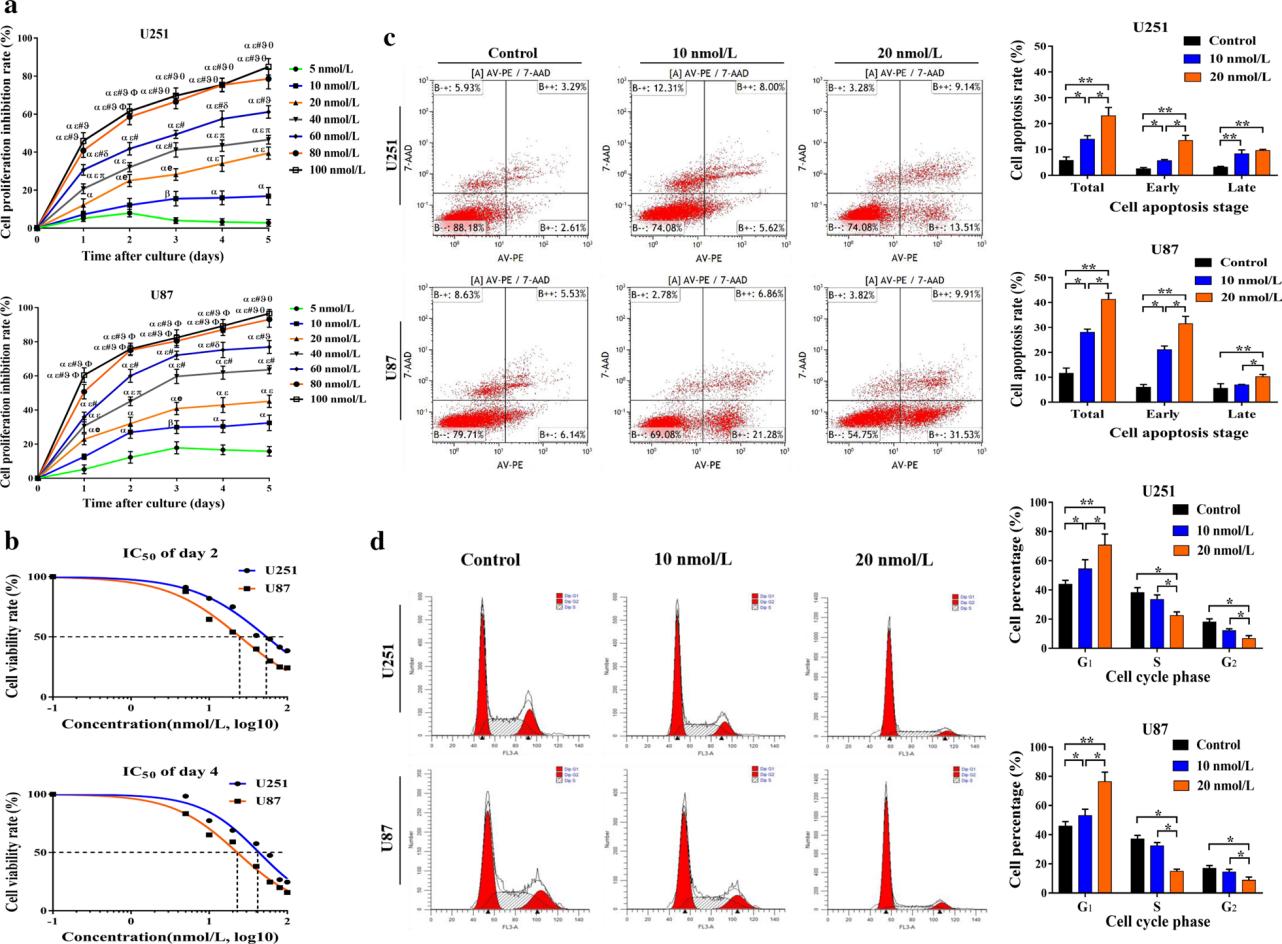
Effects of bortezomib on the proliferation, apoptosis, and cell cycle of glioma cells. **a** MTT assay measured the viability of U251 and U87 cell lines under 0 (Control, DMSO), 5, 10, 20, 40, 60, 80, and 100 nmol/L bortezomib treatment. The cell proliferation inhibition rate of each treatment group was compared with that of every other group detected on the same day. ^ɑ^*P* < 0.01, ^β^*P* < 0.05, compared with the 5 nmol/L group; ^ε^*P* < 0.01, ^e^*P* < 0.05, compared with 10 nmol/L group; ^#^*P* < 0.01, ^π^*P* < 0.05, compared with 20 nmol/L group; ^ϑ^*P* < 0.01, ^δ^*P* < 0.05, compared with 40 nmol/L group; ^θ^*P* < 0.01, ^Ф^*P* < 0.05, compared with 60 nmol/L group. **b** Day 2 and Day 4 IC_50_ of bortezomib in U251 and U87 cells were calculated with the method of “log(inhibitor) vs. normalized response-Variable slope” using GraphPad Prism 7.0. **c** Left part, representative images of cell apoptosis detected via flow cytometry. U251 and U87 cells were treated with 10 and 20 nmol/L bortezomib for 48 h. Right part, percentages of early-stage (lower right quadrant), late-stage (upper right quadrant), and total apoptotic cells were compared among the three groups. *P < 0.05; **P < 0.01. **d** Left part, representative images of cell cycle detected via flow cytometry. U251 and U87 cells were treated with 10 and 20 nmol/L bortezomib for 48 h. Right part, percentages of cells in G_0/1_ (left red sharp peak), S (middle gray flat peak), and G_2_/M (right sharp peak) phases were calculated and compared among groups. *P < 0.05; **P < 0.01. All experiments were repeated at least three times. *DMSO* dimethyl sulfoxide, *IC*_*50*_ 50% inhibitory concentration

Using the MTT assays, we observed that bortezomib inhibited the viability of glioma cells in a dose- and time-dependent manner (Fig. 1a). Even at a concentration as low as 10 nmol/L, bortezomib significantly inhibited the proliferation of both U251 and U87 cells (*P* < 0.05). When concentration decreased to 5 nmol/L, bortezomib remained cytotoxic to U87 cells, but no obvious inhibition of viability was observed in U251 cells, indicating a slight difference in bortezomib sensitivity among the glioma cell lines (Fig. 1a). For both U251 and U87 cells, the IC_50_ of day 4 were higher than that of day 2 (*P* < 0.05), indicating the time-dependent effect as well as long duration of action of bortezomib on glioma cells (Fig. 1b). By flow cytometry, we further found significant increases of apoptotic U251 and U87 cells, especially early-stage apoptotic cells, after bortezomib treatment (*P* < 0.05 or *P* < 0.01) (Fig. 1c; Table 3). Bortezomib also caused obvious cell cycle arrest in U25 and U87 cells, which was characterized by increases of G_1_ phase cells and decreases of S and G_2_ phase cells (*P* < 0.05 or *P* < 0.01) (Fig. 1d; Table 4). Compared with U251 cells, U87 cells demonstrated more severe cell apoptosis and cell cycle arrest when under the same drug concentration. This was in accordance with the intenser cytotoxicity of bortezomib in U87 as mentioned above.

**Table 3:** Cell apoptosis after bortezomib treatment

Cell line	Group	Cell apoptosis rate (%)		
		Total	Early-stage	Late-stage
U251	Control	5.78 ± 1.34	2.56 ± 0.44	3.22 ± 0.26
	10 nmol/L Bor	14.01 ± 1.33	5.68 ± 1.42	8.33 ± 1.51
	20 nmol/L Bor	23.07 ± 3.21	13.49 ± 1.97	9.58 ± 2.50
U87	Control	11.62 ± 2.12	6.02 ± 1.08	5.58 ± 1.86
	10 nmol/L Bor	28.03 ± 31.34	21.08 ± 1.52	6.95 ± 0.21
	20 nmol/L Bor	41.17 ± 2.55	31.48 ± 2.97	10.23 ± 0.93

**Table 4:** Cell cycle alteration after bortezomib treatment

Cell line	Group	Percentage of cells in different cell cycle phases (%)		
		Phase G_1_	Phase S	Phase G_2_
U251	Control	43.87 ± 2.77	38.12 ± 3.50	18.01 ± 2.23
	10 nmol/L	54.44 ± 6.32	33.42 ± 3.22	12.15 ± 1.22
	20 nmol/L	70.83 ± 7.54	22.42 ± 2.52	6.74 ± 2.10
U87	Control	45.97 ± 2.99	37.06 ± 2.52	16.97 ± 1.88
	10 nmol/L	53.1 ± 64.3	32.41 ± 2.21	14.43 ± 1.90
	20 nmol/L	76.35 ± 6.54	14.89 ± 1.50	8.76 ± 2.22

### Bortezomib inhibited spheroid growth, colony formation, and stemness of glioma cells

**Fig. 2:**
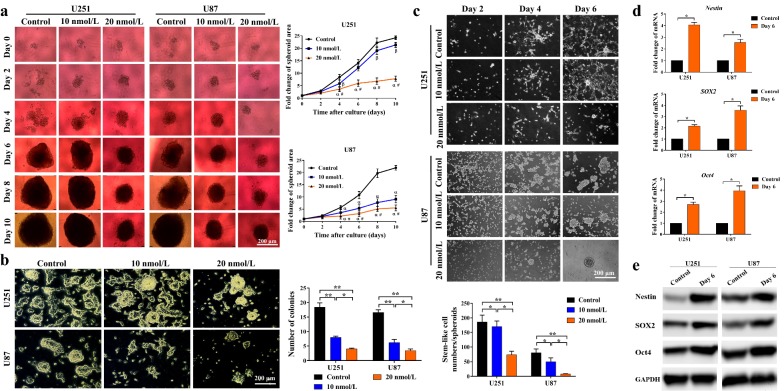
Effects of bortezomib on the spheroid growth, colony formation, and stemness of glioma cells. **a** Left part, representative images of U251 and U87 cell spheroids treated with 0 (Control), 10, and 20 nmol/L bortezomib. Right part, the growth speed is represented by the fold changes of the spheroid area compared with its own area on day 1. Images of spheroids were taken every 2 days (scale bar, 200 μm). Average fold change of the spheroid area was compared between every two groups detected on the same day. ^ɑ^*P* < 0.01, ^β^*P* < 0.05, compared with control group; ^#^*P* < 0.01, ^π^*P* < 0.05, compared with 10 nmol/L bortezomib group. **b** Bortezomib reduced glioma cell colony formation. Left part, representative images of U251 and U87 colonies on the 10th day (with phase-contrast mode, scale bar, 200 μm). Cells were cultured in medium with 0 (Control, DMSO), 10, or 20 nmol/L bortezomib for the first 3 days and then in normal medium for another 7 days. Right part, the average number of cell colonies observed under 5 random microscopic fields was calculated. **c** Upper part, representative images of stem-like cells/spheroids derived from U251 and U87 cells in stem cell culture medium (scale bar, 200 μm). Cells were treated with 0 (Control, DMSO), 10, or 20 nmol/L bortezomib for 24 h before seeding. Bottom part, the average number of stem-like cells/spheroids (more than 20 cells) observed under 5 random microscopic fields on day 6 was calculated. **d**, **e** RT-qPCR and Western blotting detected the mRNA and protein levels of cells/spheroids enriched after stem cell medium culture. *P < 0.05; **P < 0.01. All experiments were repeated at least three times. *DMSO* dimethyl sulfoxide, *RT-qPCR* reverse transcription-quantitative polymerase chain reaction, *GAPDH* glyceraldehyde-3-phosphate dehydrogenase

The tumor cell spheroid model better imitates the in vivo growth situation of glioma. Therefore, we tested the chemotherapeutic effect of bortezomib on the 3-dimensional (3D) spheroid models of U251 and U87 cells. From day 4 to day 12, fold changes of the surface area for spheroids were significantly lower in the bortezomib-treated groups than in the corresponding control groups (*P* < 0.05 or *P* < 0.01) (Fig. 2a). The difference between bortezomib treated groups and corresponding control group turned out to be more significant as time went on, and the 20 nmol/L bortezomib group consistently showed relatively smaller spheroids than the 10 nmol/L bortezomib group after the 4th day (*P* < 0.05 or *P* < 0.01), indicating dose- and time-dependent inhibitory effects of bortezomib on in vitro glioma cell spheroids. The colony formation experiment was also conducted to demonstrate the in vitro tumorigenic capacity of glioma cells. Fewer colonies were developed in U251 and U87 cells after treatment with 10 nmol/L or 20 nmol/L bortezomib for the first 3 days (*P* < 0.05 or *P* < 0.01) (Fig. 2b). Experiments were also conducted to test whether bortezomib possesses a stemness-inhibiting effect on glioma cells. Groups treated with 10 or 20 nmol/L bortezomib developed much fewer stem-like cells (U251) or float spheroids (U87) than their corresponding control cells (Fig. 2c). Compared with the control and 10 nmol/L bortezomib groups, the 20 nmol/L bortezomib group had the fewest stem-like cells (U251) or float spheroids (U87) (*P* < 0.05 or *P* < 0.01) (Fig. 2c). The mRNA and protein expression levels of Nestin, SOX2, and Oct4 were markedly up-regulated in these stem-like cells/spheroids (*P* < 0.05) (Fig. 2d, e), indicating their close similarity to GSCs and that bortezomib might inhibit the stemness of glioma cells in a dose-dependent manner.

### Bortezomib down-regulated FOXM1–Survivin axis in glioma cells

**Fig. 3:**
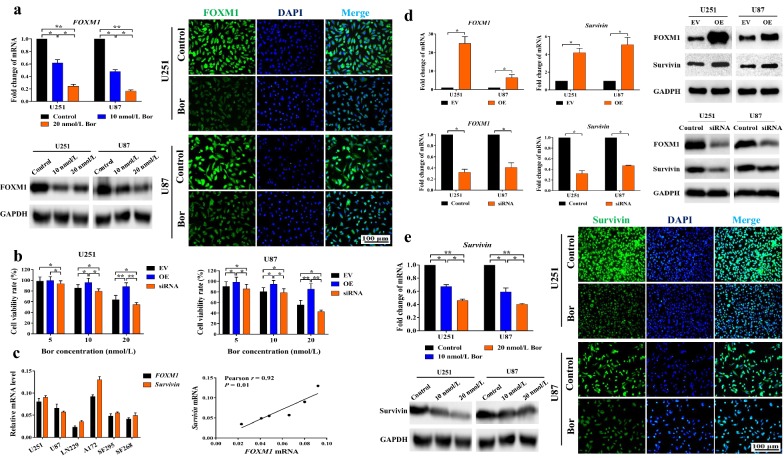
Bortezomib down-regulated the FOXM1–Survivin axis in glioma cells. **a** Left part, measuring *FOXM1* in U251 and U87 after treatment with 0 (Control, DMSO), 10, or 20 nmol/L bortezomib for 48 h at the mRNA level (RT-qPCR, left upper part) and protein level (Western blotting, left lower part). Right part, immunofluorescent staining of FOXM1 in U251 and U87 cells after bortezomib treatment for 48 h (scale bar, 100 μm). **b** Same number of *FOXM1*-overexpressed, *FOXM1* siRNA-transfected, and EV-transfected cells were treated with bortezomib for 48 h. Cell viability was measured by MTT assay. **c** *FOXM1* and *Survivin* mRNA levels in 6 glioma cell lines were detected by RT-qPCR. The correlation between *FOXM1* and *Survivin* mRNA levels was analyzed via GraphPad Prism 7.0 using Pearson *R* test. **d** *FOXM1* and *Survivin* mRNA (RT-qPCR, left part) and protein expression (Western blotting, right part) in *FOXM1*-overexpressed, *FOXM1* siRNA-transfected, and EV-transfected cells. **e** Left part, *Survivin* mRNA (RT-qPCR, left upper part) and protein expression (Western blotting, left lower part) in U251 and U87 cells after treatment with bortezomib for 48 h. Right part, immunofluorescent staining of Survivin in U251 and U87 cells after treatment with bortezomib for 48 h (scale bar, 100 μm). *P < 0.05; **P < 0.01. *RT-qPCR* reverse transcription-quantitative polymerase chain reaction, *DMSO* dimethyl sulfoxide, *GAPDH* glyceraldehyde-3-phosphate dehydrogenase, *DAPI* 4′,6-diamidino-2-phenylindole, *siRNA* short interfering RNA, *Bor* bortezomib, *EV* empty vector, *OE* overexpression, *MTT* 3-(4,5-dimethyl-2-thiazolyl)-2,5-diphenyl-2-*H*-tetrazolium bromide

The high sensitivity of glioma cells to bortezomib indicated that its targets might be critical for glioma cell survival. We found that bortezomib significantly down-regulated the mRNA level (*P* < 0.05 or *P* < 0.01), protein level, and immunofluorescence intensity of FOXM1 (Fig. 3a). To confirm whether *FOXM1* was one of the principal targets of bortezomib, we established *FOXM1* overexpression and knockdown cell lines and tested their sensitivity to bortezomib. Compared with corresponding EV-transfected cells, *FOXM1*-overexpressed cell lines had higher cell viability rates, while *FOXM1* knockdown cell lines had lower cell viability rates after 10 nmol/L and 20 nmol/L bortezomib treatment (*P* < 0.05 or *P* < 0.01) (Fig. 3b). These results showed that *FOXM1* overexpression inhibited sensitivity to bortezomib, while *FOXM1* knockdown enhanced sensitivity to bortezomib. *FOXM1* down-regulation might be the main mechanism underlying the efficient cytotoxicity of bortezomib.

To confirm whether Survivin was an important downstream effector of FOXM1, we firstly measured the mRNA levels of *FOXM1* and *Survivin* in six glioma cell lines: U251, U87, LN229, A172, SF295, and SF268. A close correlation between *FOXM1* mRNA level and *Survivin* mRNA level was indicated in them (Pearson *r *= 0.92, *P* = 0.01) (Fig. 3c). Further, *Survivin* mRNA and protein levels were significantly up-regulated by *FOXM1* overexpression and markedly reduced by *FOXM1* knockdown in U251 and U87 cells (*P* < 0.05) (Fig. 3d). In accordance with *FOXM1* alteration, the mRNA and protein levels, as well as immunofluorescent staining of Survivin were significantly reduced after bortezomib treatment (*P* < 0.05 or *P* < 0.01) (Fig. 3e). In summary, bortezomib down-regulated the FOXM1–Survivin axis in U251 and U87 cells, and this might be an important molecular mechanism of its chemotherapeutic effects, alone or in combination with other agents.

### Bortezomib sensitized glioma cells to TMZ

**Fig. 4:**
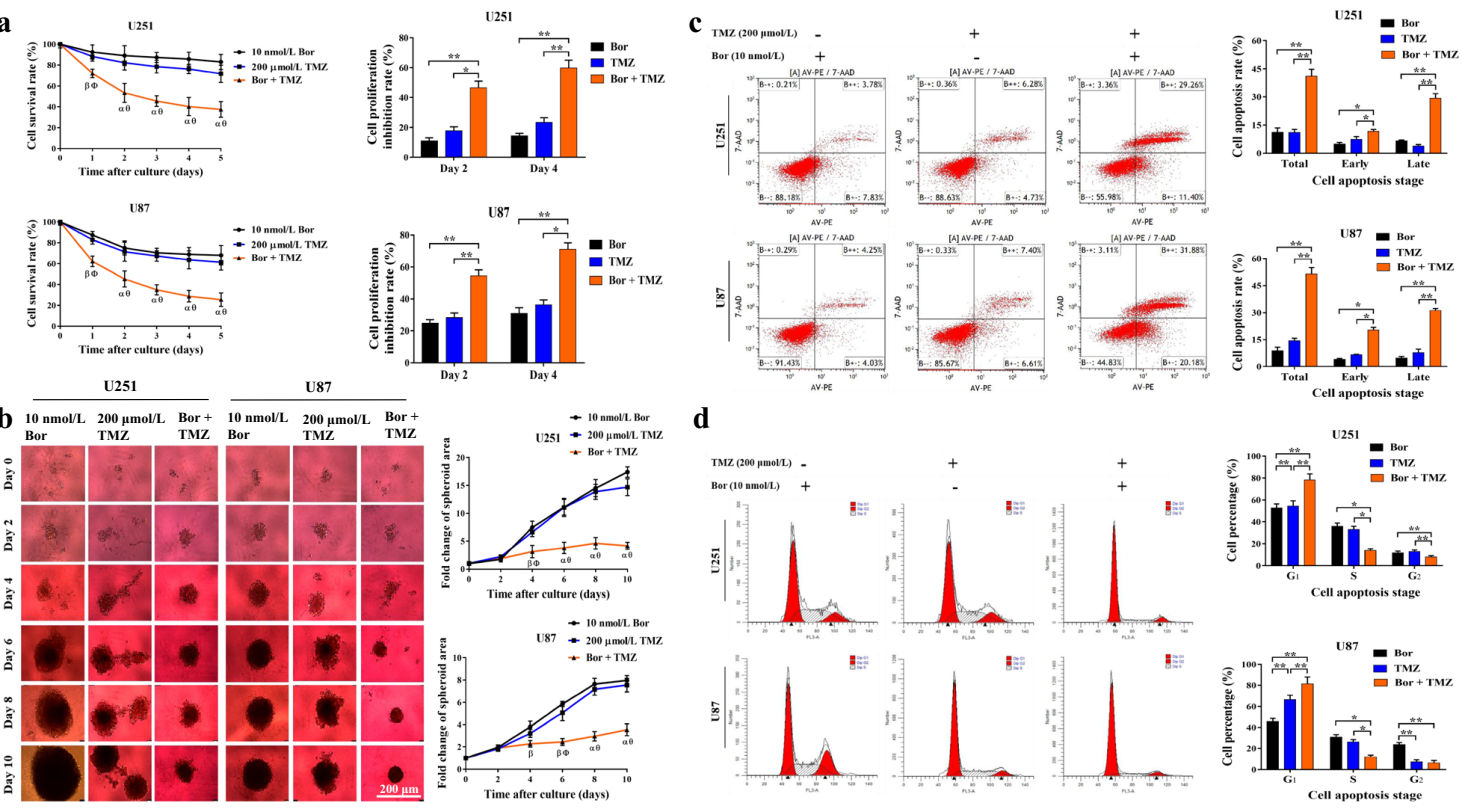
Bortezomib sensitized glioma cells to TMZ. U251 and U87 cells were treated with bortezomib (10 nmol/L), TMZ (200 μmol/L) or a combination of the two drugs. **a** Left part, viability of U251 and U87 cells was measured by MTT assay. Cell survival rates were compared among groups. Right part, on day 4 and day 2, cell proliferation inhibition rates were calculated. ^ɑ^*P* < 0.01, ^β^*P* < 0.05, compared with 10 nmol/L bortezomib group; ^θ^*P* < 0.01, ^Ф^*P* < 0.05, compared with 200 μmol/L TMZ group; *P < 0.05; **P < 0.01. **b** Left part, representative images of U251 and U87 spheroids taken every 2 days (scale bar, 200 μm). Right part, growth speed represented by the fold change of surface area compared with the surface area on day 1. Fold changes (in average) from the same day were compared among the three groups. ^ɑ^*P* < 0.01, ^β^*P* < 0.05, compared with 10 nmol/L bortezomib group; ^θ^*P* < 0.01, ^Ф^*P* < 0.05, compared with 200 μmol/L TMZ group. **c** Left part, representative images of cell apoptosis after 48-h treatment detected via flow cytometry. Right part, percentages of early-stage (lower right quadrant) and late-stage (upper right quadrant) apoptotic cells and their sum were compared among the three groups. **d** Left part, representative images of cell cycle detected via flow cytometry after 48-h treatment. Right part, percentages of cells in G_0/1_ (left red sharp peak), S (middle gray flat peak), and G_2_/M (right sharp peak) phase were calculated and compared among groups. *P < 0.05; **P < 0.01. All experiments were repeated at least three times. *MTT* 3-(4,5-dimethyl-2-thiazolyl)-2,5-diphenyl-2-*H*-tetrazolium bromide, *DMSO* dimethyl sulfoxide, *Bor* bortezomib, *TMZ* temozolomide

Searching molecular inhibitors that have a synergistic effect with TMZ is an important strategy to reduce drug resistance and improve TMZ efficacy. MTT results showed that the combination index of 200 μmol/L TMZ with different concentrations of bortezomib was consistently below “1”, indicating a synergistic effect between TMZ and bortezomib (Table 5). For instance, the combination of bortezomib (10 nmol/L) and TMZ (200 μmol/L) led to markedly lower cell survival rates and significantly higher proliferation inhibition rates than bortezomib and TMZ alone (*P* < 0.05 or *P* < 0.01) (Fig. 4a). This synergistic effect in inhibiting glioma cell viability was further confirmed by 3D tumor spheroid assay. Compared with the bortezomib group and the TMZ group, the combination group had significantly lower surface area fold changes since day 4 (*P* < 0.05 or *P* < 0.01) (Fig. 4b). In line with observations of cell viability and spheroid growth, flow cytometry showed that the combination treatment caused much higher cell apoptosis rates than single drug treatment (Fig. 4c). Furthermore, in both U251 and U87 cell lines, cell apoptosis rates induced by the combination treatment were higher than those caused by bortezomib and TMZ alone (*P* < 0.05 or *P* < 0.01) (Fig. 4c; Table 6). These results indicated that a synergistic effect might exist between bortezomib and TMZ for inducing tumor cell apoptosis. Cell cycle assay also showed that the combination treatment caused more obvious alteration in cell cycle than bortezomib and TMZ did, although all caused an increase of cells in G_1_ phase and reduction of cells in S and G_2_ phases (*P* < 0.05 or *P* < 0.01) (Fig. 4d; Table 7).

**Table 5:** Combination index of the temozolomide (TMZ) in combination with different concentrations of bortezomib (Bor)

Bor	U251		U87	
Dose (nmol/L)	Effect	Combination index	Effect	Combination index
2	0.24	0.74	0.33	0.67
5	0.32	0.64	0.42	0.61
8	0.38	0.60	0.58	0.44
10	0.47	0.50	0.61	0.44
15	0.71	0.29	0.71	0.38
20	0.85	0.19	0.85	0.23

TMZ was used at a constant dose of 200 μmol/L. Effect: cell viability inhibition rate

**Table 6:** Cell apoptosis after treatment with bortezomib (Bor) and temozolomide (TMZ)

Cell line	Group	Cell apoptosis rate (%)		
		Total	Early-stage	Late-stage
U251	20 nmol/L Bor	11.23 ± 2.22	4.75 ± 0.92	6.48 ± 0.51
	200 μmol/L TMZ	11.06 ± 2.63	7.26 ± 1.52	3.80 ± 0.76
	Bor + TMZ	40.96 ± 3.88	11.68 ± 0.97	29.28 ± 2.56
U87	20 nmol/L Bor	8.84 ± 1.99	4.06 ± 0.54	4.78 ± 0.88
	200 μmol/L TMZ	14.52 ± 1.36	6.68 ± 0.25	7.84 ± 1.92
	Bor + TMZ	51.52 ± 3.59	20.33 ± 2.58	31.22 ± 2.18

**Table 7:** Cell cycle alteration after treatment with bortezomib (Bor) and temozolomide (TMZ)

Cell line	Group	Percentage of cells in different cell cycle phases (%)		
		G_1_	S	G_2_
U251	20 nmol/L Bor	52.5 ± 3.99	35.98 ± 2.96	11.52 ± 1.66
	200 μmol/L TMZ	54.45 ± 4.88	32.77 ± 3.25	12.78 ± 1.42
	Bor + TMZ	78.31 ± 5.54	13.83 ± 1.58	7.86 ± 1.26
U87	20 nmol/L Bor	45.67 ± 2.99	30.73 ± 2.53	23.63 ± 1.88
	200 μmol/L TMZ	66.44 ± 4.33	26.19 ± 2.20	7.37 ± 1.94
	Bor + TMZ	81.40 ± 6.51	12.22 ± 1.54	6.42 ± 2.25

### FOXM1–Survivin axis was up-regulated in TMZ-insensitive glioma cell lines

**Fig. 5:**
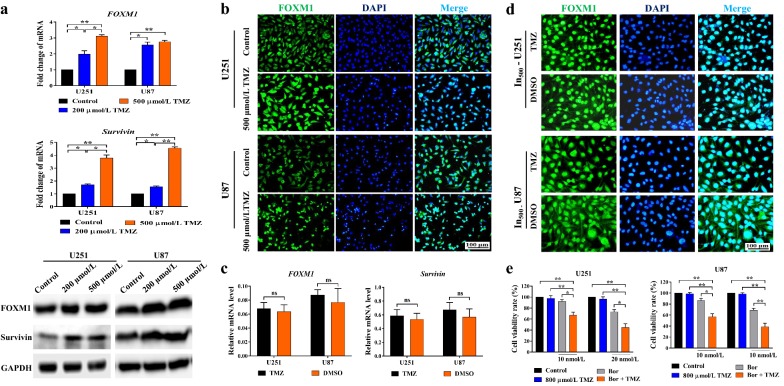
FOXM1–Survivin axis was up-regulated in TMZ-insensitive glioma cells. **a** RT-qPCR (mRNA level, upper part) and Western blotting (protein level, lower part) measuring the expression of *FOXM1* and *Survivin* in TMZ-insensitive U251 and U87 cells induced by 200 μmol/L and 500 μmol/L TMZ (In_200_-U251/U87 and In_500_-U251/U87). **b** Immunofluorescent staining of FOXM1 in normal (Control) and In_500_-U251/U87 cells (scale bar, 100 μm). **c** RT-qPCR detecting mRNA levels of *FOXM1* and *Survivin* in In_500_-U251/U87 cells after cultivating in medium with 500 μmol/L TMZ or equivalent DMSO for 7 days. **d** Immunofluorescent staining of FOXM1 protein in In_500_-U251/U87 cells after cultivating in medium with 500 μmol/L TMZ or equivalent DMSO for 7 days (scale bar, 100 μm). **e** Cell viability rates were calculated after In_500_-U251/U87 cells were treated with 10 or 20 nmol/L bortezomib, 800 μmol/L TMZ, bortezomib + TMZ, or drug vehicle (DMSO, control) for 48 h. *P < 0.05; **P < 0.01. *RT-qPCR* reverse transcription-quantitative polymerase chain reaction, *TMZ* temozolomide, *DAPI* 4′,6-diamidino-2-phenylindole, *DMSO* dimethyl sulfoxide, *Bor* bortezomib, *ns* no significance

Validating the role of the FOXM1–Survivin axis in TMZ resistance will further strengthen the evidence for combination use of bortezomib and TMZ. We established TMZ-insensitive U251 and U87 cell lines (In-U251 and In-U87) and found that they had higher *FOXM1* and *Survivin* expression (in both mRNA and protein levels) than their corresponding control cell lines (*P* < 0.05 or *P* < 0.01) (Fig. 5a). The up-regulated protein level of FOXM1 in 500 μmol/L TMZ-induced insensitive cell lines was further confirmed by using immunofluorescent staining (Fig. 5b). In addition, the mRNA levels of *FOXM1* and *Survivin* and immunofluorescent staining of FOXM1 in TMZ-insensitive cell lines were not significantly altered after culture in normal medium for 7 days, indicating relatively stable up-regulation of *FOXM1* and *Survivin* in TMZ-insensitive cell lines (Fig. 5c, d). On the other hand, most survived clones might be highly dependent on the FOXM1–Survivin axis for proliferation and survival, making them quite susceptible to reagents especially targeting FOXM1 and Survivin. In validating this hypothesis, 500 μmol/L TMZ-induced insensitive U251 and U87 cell lines (In_500_-U251 and In_500_-U87) were treated with bortezomib, TMZ, or their combination. No significant alteration of cell viability was caused by TMZ treatment, while dramatically reduced cell viability was detected in all combination groups, suggesting that synergistic inhibitory effect of bortezomib and TMZ still worked in highly TMZ-insensitive glioma cell lines (*P* < 0.05 or *P* < 0.01) (Fig. 5e).

### Bortezomib inhibited glioma growth and enhanced TMZ efficacy in vivo

**Fig. 6:**
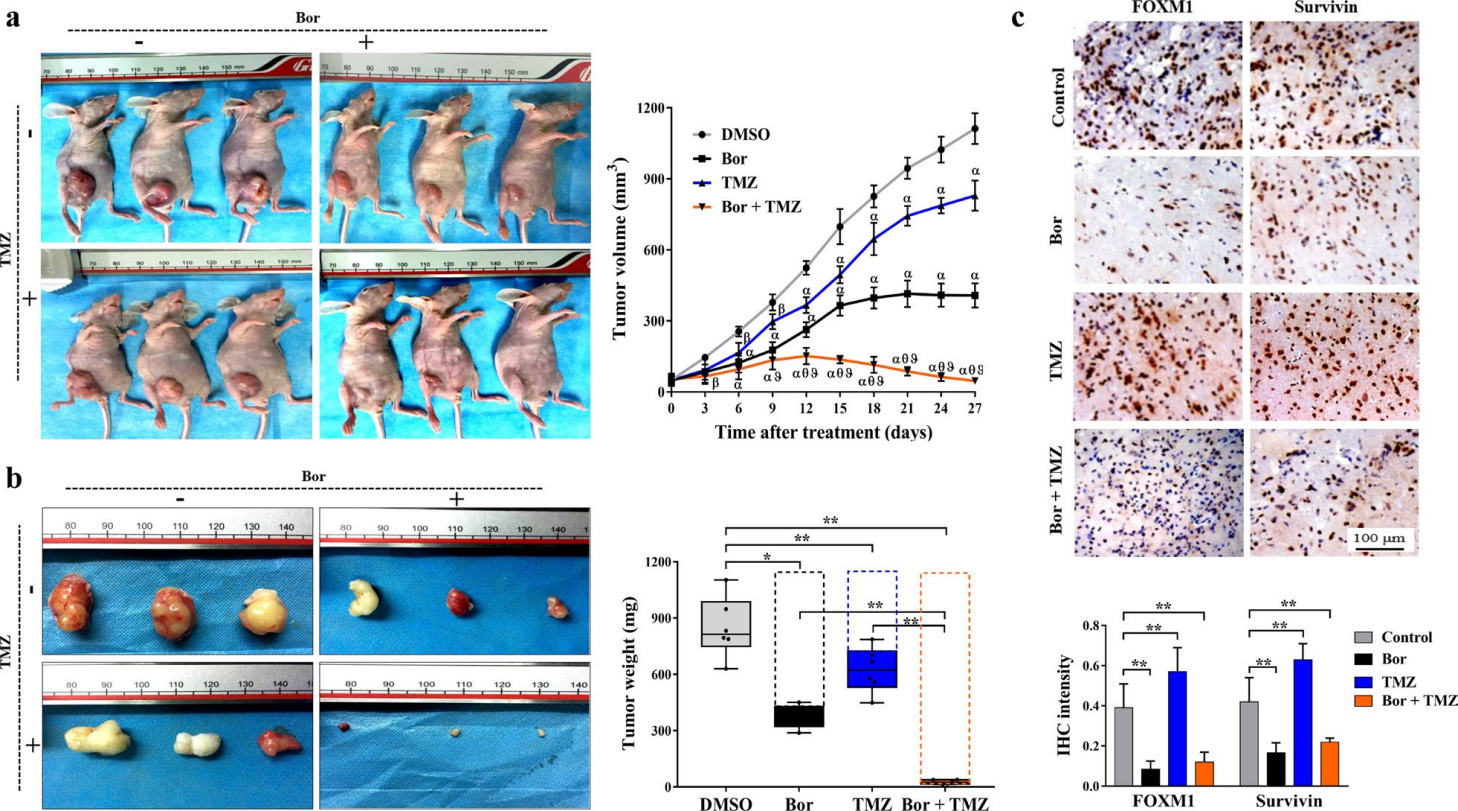
Bortezomib inhibited glioma growth and sensitized glioma to TMZ in vivo. **a** Left part, representative images of subcutaneously xeno-transplanted glioma models in nude mice. About 1 week after subcutaneous injection of U87 cells, the mice were selected and randomized into four groups and were initiated treatment with bortezomib, TMZ, TMZ + bortezomib, or drug vehicle (DMSO). Right part, in vivo tumors volume was measured every 3 days with a vernier caliper, and same day collected data were compared among groups. ^ɑ^*P* < 0.01, ^β^*P* < 0.05, compared with DMSO group; ^θ^*P* < 0.01, compared with bortezomib group; ^ϑ^*P* < 0.01, compared with TMZ group. **b** Left part, representative images of glioma lesions taken from the mice of each group. After treatment for 28 days, the nude mice were euthanized, and glioma lesions were taken off in intact. Right part, the weight of fresh glioma lesions. **c** Top part, representative images of IHC staining of FOXM1 and Survivin in glioma tissues from mice (scale bar, 100 μm). Bottom part, the IHC staining intensity of FOXM1 and Survivin were further quantified via Image-pro Plus 6.0. *P < 0.05; **P < 0.01. *Bor* bortezomib, *TMZ* temozolomide, *DMSO* dimethyl sulfoxide, *IHC* immunohistochemistry

The in vivo chemotherapeutic effect of bortezomib was further explored by xenograft glioma models in nude mice. Compared with the control group, the bortezomib and TMZ groups had much smaller tumor sizes since day 6 (after treatment initiation), and this difference became more obvious with time. Average tumor volume of the combination group was strikingly smaller than both the bortezomib and TMZ groups since day 9 (*P* < 0.05 or *P* < 0.01) (Fig. 6a). In fact, the combination treatment even achieved complete tumor regression in some mice. Similar to the alteration in tumor volume, the mean tumor weights (at the end of treatment) in the bortezomib and TMZ groups were much smaller than that of the control group and significantly larger than that of the combination group (*P* < 0.05 or *P* < 0.01) (Fig. 6b). These results demonstrated that a low concentration of bortezomib could efficiently inhibit the in vivo growth of U87 cells, and the combination of bortezomib and TMZ exerted a much stronger growth-inhibitory effect on U87 xenograft models. Comparing with those in the corresponding control group, the IHC intensities of both FOXM1 and Survivin were markedly increased in the TMZ group and substantially decreased in the bortezomib group and the combination group (*P* < 0.05 or *P* < 0.01) (Fig. 6c), indicating the possible in vivo down-regulating effect of bortezomib on the FOXM1–Survivin axis.

### The FOXM1–Survivin axis was up-regulated in glioma and related to poor prognosis

**Fig. 7:**
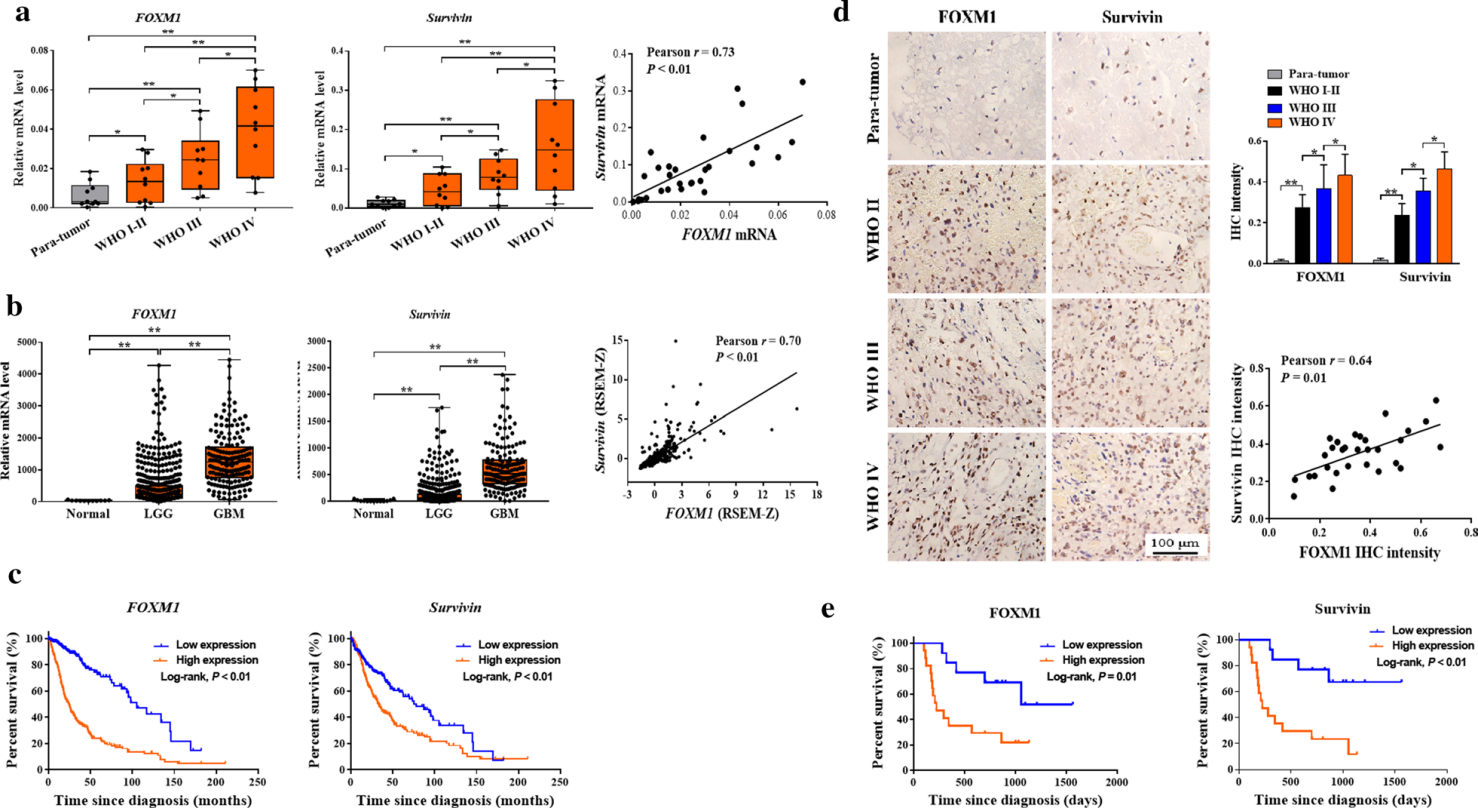
The FOXM1–Survivin axis was up-regulated in gliomas and related to poor prognosis. **a** RT-qPCR measuring mRNA levels of *FOXM1* and *Survivin* in para-tumor brain tissues (*n* = 10), WHO grade I–II gliomas (*n *= 10), WHO grade III gliomas (*n* = 10), and WHO grade IV gliomas (GBMs, *n* = 10). The correlation between *FOXM1* and *Survivin* mRNA levels was analyzed via GraphPad Prism 7.0 using Pearson *R* test. **b** TCGA data of *FOXM1*/*Survivin* mRNA expression (mRNA Expression z-Scores, RNA SeqV2 RSEM) in LGGs (*n* = 530), GBMs (*n* = 166), and normal brain tissues (*n *= 10). The correlation between *FOXM1* and *Survivin* mRNA levels (696 samples in total) was analyzed using GraphPad Prism 7.0 and Pearson *R* test. **c** Kaplan–Meier survival analysis of the prognostic role of *FOXM1*/*Survivin* using TCGA data (692 samples in total). The samples were divided into a high and low *FOXM1/Survivin* expression group using their relative median mRNA level. **d** Left part, representative images of IHC staining of FOXM1 and Survivin in para-tumor brain tissues and gliomas (scale bar, 100 μm). Right part, IHC intensity was measured and processed by Image-pro Plus 6.0. The correlation between FOXM1 and Survivin IHC intensity was analyzed using GraphPad Prism 7.0 and Pearson *R* test. **e** Kaplan–Meier survival analysis of the prognostic role of FOXM1 and Survivin. The samples were divided into high and low FOXM1/Survivin expression groups by the median IHC intensity. *P < 0.05; **P < 0.01. *RT-qPCR* reverse transcription-quantitative polymerase chain reaction, *PT* para-tumor brain tissue, *WHO* World Health Organization, *LGG* low-grade glioma, *GBM* glioblastoma multiforme, *TCGA* The Cancer Genome Atlas, *RSEM* RNA-seq by expectation maximization, *IHC* immunohistochemistry

The up-regulation of the FOXM1–Survivin axis in clinical samples can, at least partially, confirm our results and indicated the potentiality of bortezomib in glioma chemotherapy. Initially, we measured the mRNA levels of *FOXM1* and *Survivin* in clinical samples of different WHO grades. Compared with those in para-tumor brain tissues, both *FOXM1* and *Survivin* mRNA levels were up-regulated in gliomas, with increasing levels in higher WHO grade gliomas (*P* < 0.05 or *P* < 0.01). The mRNA levels of *FOXM1* and *Survivin* were positively correlated with each other (*r* = 0.73, *P* < 0.01) (Fig. 7a). In accordance with our results in clinical samples, TCGA data also demonstrated much higher *FOXM1* and *Survivin* mRNA levels in GBM and low-grade glioma (LGG) than in normal brain tissues (*P* < 0.05 or *P* < 0.01), and the correlation between them was positive and significant in both LGG and GBM (*r* = 0.70, *P* < 0.01) (Fig. 7b). TCGA data also showed shorter overall survival (OS) in patients with higher *FOXM1*/*Survivin* mRNA levels (*P* < 0.01) (Fig. 7c). To further confirm the *FOXM1* up-regulation in protein level, FOXM1 and Survivin protein levels were detected by IHC in all our gliomas and PT brain tissues. Compared with those in para-tumor brain tissues, FOXM1 and Survivin IHC staining intensities were markedly increased in tumor tissues, especially in GBMs (*P* < 0.05 or *P* < 0.01). In line with the positive correlation in mRNA level, IHC staining intensity of Survivin was also positively correlated with that of FOXM1 (*r* = 0.64, *P* = 0.01) (Fig. 7d). Furthermore, we found that patients with higher FOXM1/Survivin intensity had a shorter OS (*P* = 0.01/*P* < 0.01), indicating the possible use of FOXM1, Survivin, or both together as prognostic molecular markers (Fig. 7e).

## Discussion

Bortezomib, a novel boronic acid dipeptide that inhibits the 26S proteasome activity, has already shown some chemotherapeutic effects against gliomas in vitro and in vivo [18–20]. Consistent with the findings by Yin et al. [21], our study demonstrated a significant decrease in cell survival and an increase in cell apoptosis in GBM cell lines treated with bortezomib at a concentration as low as 10 nmol/L (Fig. 1a, c). We also found that a low concentration of bortezomib significantly inhibited spheroid growth, colony formation, and stem-like cell proliferation of U251 and U87 cells (Fig. 2a–c). GSCs are defined as a highly tumorigenic cell subset responsible for tumor progression and drug resistance and is expected to be a critical therapeutic target in various malignancies [22, 23]. Interestingly, recent studies have shown that GSCs were more sensitive to PIs than non-stem differentiated controls or neural stem/precursor cells [24, 25]. Thus, the chemotherapeutic effect of bortezomib on HGG cell lines might result from the specific deletion of GSCs, or at least the GSC sub-population was preferentially inhibited/killed by bortezomib.

In regard to the molecular mechanism underlying the chemotherapeutic effect of bortezomib on glioma cells, generally, it is assumed that the pharmacological inhibition of the proteasome led to toxic accumulation of misfolded and abnormal proteins in cells [26]. For instance, several studies have reported reduced activation of nuclear factor-kappa B after inhibiting IκB degradation by PIs [27]. P53 has been also widely studied in exploring the molecular mechanism of PI-induced tumor apoptosis [28]. Many studies have demonstrated that proteasome inhibition led to growth suppression and apoptosis of tumor cells via inducing overwhelming endoplasmic reticulum stress [29]. Some other possible mechanisms have also been proposed, but the exact mechanism by which PIs exert anti-tumor activity is still poorly understood, especially in glioma. In the present study, we found that bortezomib treatment led to a significant reduction in both mRNA and protein expression of FOXM1 (Fig. 3a), which is very important for tumor malignant behaviors [30]. In support of our findings, similar results were also reported by Andrei L. Gartel and colleagues. They initially found two novel PIs (Siomycin A and thiostrepton), then a serial of conventional PIs that could down-regulate not only the transcriptional activity but also the protein and mRNA levels of *FOXM1* [31–33]. The *FOXM1* auto-regulation loop, whereby FOXM1 binds to its own promoter and induces its own transcription [34], seems to be a feasible mechanism for PI-induced alteration in both protein and mRNA levels. In our present study, another oncogene, the anti-apoptotic factor *Survivin*, was found to be regulated by *FOXM1* in glioma cells (Fig. 3d, e). FOXM1 has been found to directly regulate the transcription of *Survivin* and the X-linked inhibitor of apoptosis protein in breast cancer cells, providing good support to our findings [35].

The present study demonstrated that down-regulating the FOXM1–Survivin axis could be an important molecular mechanism for using bortezomib in treating gliomas. However, more details involving bortezomib and FOXM1 interaction await clarification. Bortezomib and other PIs may change the activity of FOXM1 directly or indirectly. In regard to direct action, bortezomib or its metabolic derivatives could attach to the substructure of FOXM1 protein and attenuate or abrogate its function, or intervene in the DNA-binding activity of the FOXM1 domain [10]. As to indirect action, even more possibilities exist. As the activity of FOXM1 protein is strictly regulated by post-translation modification, certain kinds of modification may make the protein lost its initial function and be a “disturber” of normal protein activity. The principle biological function of PIs is repressing the function of the proteasome system [36]. Bortezomib might cause accumulation of this “disturbing” FOXM1 and subsequently attenuated DNA-binding and transcriptional activity of normal FOXM1 [37]. In agreement to this “inhibiting” hypothesis, Andrei L. Gartel [38] proposed the “negative regulator of *FOXM1* (NRFM)” theory: the expression of a putative NRFM is accumulated after treatment with PIs then, this NRFM binds to FOXM1 and inhibits its transcriptional activity on the promoter of target genes, including *FOXM1*. Although several tumor suppressors, such as heat shock protein 70 and sterile alpha motif-pointed domain-containing E26 transformation-specific transcription factor, were found to suppress *FOXM1* auto-regulation by inhibiting the activity of *FOXM1* binding to its own gene promoter [39, 40], “NRFM” remains to be verified. Contrary to the above discussed “accumulation” effects by inhibiting proteasome activity, bortezomib may also accelerate the “reduction” of proteins by inducing autophagy/lysosomal degradation, which was reported in various tumor cells, including glioma cell lines [41–44]. Interestingly, the degradation of FOXM1 partially depends on lysosome under normal conditions [45]. Thus, bortezomib might reduce FOXM1 by enhancing lysosomal degradation of its protein. Another less possible mechanism is the down-regulation of “positive regulator of *FOXM1*”, such as transcription factors for FOXM1. Again, not much has been revealed about this hypothesis.

As summarized in a review by Hanahan et al. [46], FOXM1 is implicated in most hallmarks of cancer. In regard to glioma, FOXM1, as an oncogenic transcription factor, also plays important roles in glioma progression and maintenance of GSC characteristics [47–49]. Studies have also reported that FOXM1 promoted glioma resistance to TMZ chemotherapy via regulating DNA damage repair gene *Rad51* and replication factor C5 [50, 51]. As one of the classic anti-apoptotic proteins, Survivin plays important role in glioma progression, recurrence, and chemoradiotherapy resistance [52–54]. In our TMZ-insensitive U251 and U87 glioma cell lines, the expression levels of *FOXM1* and *Survivin* were markedly up-regulated (Fig. 5a, b). This might have resulted from the enrichment of clones with high levels of *FOXM1* and *Survivin*, while clones with lower *FOXM1* and *Survivin* expression were deleted by continuous TMZ screening. Thus, a highly activated FOXM1–Survivin axis could greatly promote glioma cell proliferation and malignant transformation, and enhance resistance and survival of glioma cells under stress.

However, the high dependence on the FOXM1–Survivin axis may make glioma cells very susceptible to agents specifically targeted to this oncogenic axis, such as bortezomib. As such, bortezomib can significantly enhance the sensitivity of glioma cells to TMZ treatment both in vitro (Fig. 4a–d) and in vivo (Fig. 6a–c). Interestingly, via analyzing clinical samples from our department and public datasets from TCGA [16], we found that *FOXM1* and *Survivin* were widely overexpressed in glioma patients (Fig. 7a, b, d). On one hand, extensive up-regulation of the FOXM1–Survivin axis in HGG patients might be the basis of possible clinical use of bortezomib in the future; on the other hand, the activation level of the FOXM1–Survivin axis in certain patients could be a good predictor for their sensitivity to bortezomib, alone or in combination with TMZ. However, several problems have to be tackled before the use of bortezomib and other PIs in glioma patients, such as the drug ability to penetrate the blood–brain barrier, unspecific targeting, and dose limited toxicity of drug [55, 56]. Further investigation is needed to further enhance the clinical use of bortezomib and to develop next-generation PIs with more satisfying clinical efficacy with advanced sensitivity, specificity, and safety [57, 58].

## Conclusions

In summary, our findings showed that PI bortezomib had a sensitive chemotherapeutic effect on glioma cells. Low concentrations of bortezomib significantly inhibited the proliferation, spheroid growth, colony formation, and stem cell characteristics of glioma cells by inducing apoptosis and cell cycle arrest. Bortezomib also demonstrated a synergistic effect with TMZ and sensitized glioma to TMZ treatment in vitro and in vivo. Mechanistically, bortezomib down-regulated the FOXM1–Survivin axis, which was also found to be up-regulated in glioma patients and was related to poor prognosis. Our findings provide an important research basis for instigating further investigation on bortezomib or other PIs in glioma therapy.

## Supplementary information

**Additional file 1: Table S1.** Antibodies for immunofluorescent, Western blotting, and immunohistochemical staining.

## Data Availability

All data (including Table 1 but not data containing patients’ other clinical information) were generated or analyzed during this study are included in this published article and its additional files. More clinical information about patients are only available from the corresponding author on reasonable request.

## Abbreviations

HGG: high-grade glioma
MTT: 3-(4,5-dimethyl-2-thiazolyl)-2,5-diphenyl-2-*H*-tetrazolium bromide
TMZ: temozolomide
FOXM1: forkhead box M1
PI: proteasome inhibitor
GSCs: glioma stem cells
OE: overexpression
EV: empty vector
siRNA: short interfering RNA
RT-qPCR: reverse transcription-quantitative polymerase chain reaction
cDNA: complementary DNA
GAPDH: glyceraldehyde-3-phosphate dehydrogenase
HRP: horseradish peroxidase
DAPI: 4′,6-diamidino-2-phenylindole
IHC: immunohistochemistry
GBM: glioblastoma multiforme
FDA: Food and Drug Administration
ATCC: American Type Culture Collection
DMEM: Dulbecco’s modified Eagle’s medium
WHO: World Health Organization
TCGA: The Cancer Genome Atlas
OD: optical density
IC_50_: 50% inhibitory concentration
PBS: phosphate buffer saline
DMSO: dimethyl sulfoxide
ANOVA: analysis of variance
3D: 3 dimensional
LGG: low-grade glioma
OS: overall survival
NRFM: negative regulator of FOXM1
