# Supplementary material for: Bortezomib inhibits growth and sensitizes glioma to temozolomide (TMZ) via down-regulating the FOXM1–Survivin axis
Source: Cancer Commun (Lond). 2019 Dec 3;39:81. doi: 10.1186/s40880-019-0424-2 (PMC6892143; doi:10.1186/s40880-019-0424-2)
Supplement: Supplementary file 1 — Additional file 1: Table S1. Antibodies for immunofluorescent, Western blotting, and immunohistochemical staining. [file 40880_2019_424_MOESM1_ESM.docx]

**Additional material:**

Supplementary Table 1. Antibodies for immunofluorescent, Western blotting, and immunohistochemical staining.

| **Antibody** | **Manufacturer** | **Concentration** | **Experiment** |
| --- | --- | --- | --- |
| **Primary** |  |  |  |
| Rabbit anti-FOXM1 monoclonal antibody [EPR17379] | Abcam | 1:100 | Immunofluorescence |
|  |  | 1:1000 | Western blotting |
|  |  | 1:250 | IHC |
| Rabbit anti-Survivin monoclonal antibody [EP2880Y] | Abcam | 1:150 | Immunofluorescence |
|  |  | 1:1000 | Western blotting |
|  |  | 1:300 | IHC |
| Mouse anti-Nestin monoclonal antibody | Beyotime Biotechnology | 1:100 | Western blotting |
| Rabbit anti-Oct4 monoclonal antibody | Beyotime Biotechnology | 1:500 | Western blotting |
| Rabbit anti-Sox2 (D6D9) monoclonal antibody #3579 | Cell Signaling Technology | 1:500 | Western blotting |
| Rabbit anti-GAPDH (14C10) monoclonal antibody #3683 | Cell Signaling Technology | 1:1000 | Western blotting |
| **Secondary** |  |  |  |
| Goat anti-rabbit IgG H&L (Alexa Fluor® 488) (ab150077), | Abcam | 1:100 | Immunofluorescence |
| Goat anti-rabbit IgG H&L (HRP) (ab205718), | Abcam | 1:2000 | Western blotting |
|  |  | 1:1500 | IHC |
| Goat anti-mouse IgG(H+L), HRP-labeled | Beyotime Biotechnology | 1:2000 | Western blotting |
| IHC: immunohistochemistry; HRP: horseradish peroxidase. Abcam, Cambridge, United Kingdom; Beyotime Biotechnology, Shanghai, China; Cell Signaling Technology, London, United Kingdom. | | | |
